# Prognostic factors affecting survival and recurrence in patients undergoing curative surgery for rectal cancer: a 10-year retrospective cohort study from a regional tertiary center in Türkiye

**DOI:** 10.3389/fonc.2025.1679585

**Published:** 2026-01-26

**Authors:** Merve Yumak, Mehmet Salim Demir

**Affiliations:** 1 Department of Gastointestinal Surgery, Van Training and Research Hospital, Van, Türkiye; 2 Department of Medical Oncology, Van Training and Research Hospital, Van, Türkiye

**Keywords:** CEA, cox regression, lymphovascular invasion, neoadjuvant therapy, perineural invasion, rectal cancer, recurrence, surgical oncology

## Abstract

**Background:**

Rectal cancer remains a major health burden worldwide, with significant morbidity and mortality despite advances in multimodal treatment. Identifying factors associated with postoperative mortality and recurrence is crucial for improving long-term outcomes.

**Objective:**

To evaluate clinical, pathological, and treatment-related predictors of overall survival (OS) and disease-free survival (DFS) in patients who underwent curative surgery for rectal cancer at a regional tertiary care center in Türkiye.

**Methods:**

This retrospective cohort study included 122 patients who underwent rectal cancer surgery between 2013 and 2023 at Van Training and Research Hospital. Demographic, clinical, and pathological variables were recorded, including tumor location, differentiation, lymphovascular invasion (LVI), perineural invasion (PNI), neoadjuvant/adjuvant therapy, and resection margin status. Survival analysis was performed using the Kaplan–Meier method, and independent prognostic factors were identified through multivariate Cox regression.

**Results:**

The median overall survival was 156.0 months (95% CI, 132.4–179.6), and the median disease-free survival was 28.5 months (95% CI, 22.0–36.5). Mortality was significantly associated with LVI (p=0.001), PNI (p=0.007), poor differentiation (p<0.001), R1 resection (p=0.013), emergency surgery (p=0.043), and follow-up metastasis (p<0.001). Patients with LVI had a 3.89-fold increased mortality risk, while follow-up metastasis increased mortality risk 8.75-fold. Recurrence was significantly associated with mid-rectal tumors, advanced T/N stage, LVI, PNI, and positive margins. Elevated carcinoembryonic antigen (CEA) levels were also predictive of poor outcomes.

**Conclusion:**

LVI, PNI, tumor grade, margin status, and follow-up metastasis are strong predictors of recurrence and mortality in rectal cancer surgery. Incorporating these parameters into postoperative risk stratification may enhance surveillance and therapeutic strategies, especially in regional healthcare settings.

## Introduction

Colorectal cancer is the third most commonly diagnosed cancer and the second leading cause of cancer-related deaths worldwide (1). Rectal cancer, which accounts for approximately one-third of colorectal malignancies, poses specific diagnostic and therapeutic challenges due to its anatomical location and biological behavior (2). In recent years, multidisciplinary management of rectal cancer has significantly evolved, improving both survival rates and local control (3).

Surgical resection remains the cornerstone of curative treatment in rectal cancer (4). However, high recurrence rates and metastatic progression continue to impact long-term outcomes despite advances in surgical techniques and adjuvant therapies (5). The introduction of total mesorectal excision (TME) and standardized oncologic resections has led to a notable decline in local recurrence (6).

Neoadjuvant chemoradiotherapy (CRT) or total neoadjuvant therapy (TNT) has been shown to improve local control, facilitate sphincter preservation, and induce pathologic complete response (pCR) in a subset of patients (7). Achieving a pCR is associated with improved disease-free and overall survival, although not all patients respond equally to neoadjuvant treatment (8). Therefore, the identification of reliable prognostic markers is crucial in personalizing treatment strategies.

Lymphovascular invasion (LVI) and perineural invasion (PNI) are histopathological features that reflect aggressive tumor biology and are associated with increased recurrence and reduced survival (9, 10). Similarly, pathologic stage, tumor differentiation, margin status, and nodal involvement are well-established prognostic indicators (11). Despite curative resection, a considerable proportion of patients still experience disease recurrence, underlining the need for improved risk stratification.

Carcinoembryonic antigen (CEA) is a widely used tumor marker in colorectal cancer, both for baseline risk estimation and surveillance during follow-up (12). Elevated preoperative and postoperative CEA levels have been linked to increased recurrence and mortality (13). The role of CEA in predicting outcomes after rectal cancer surgery remains an area of ongoing investigation.

Although several prognostic models have been proposed, variability in individual outcomes persists, especially among patients with similar staging profiles (14). Real-world data from tertiary centers can provide valuable insights into the complex interplay of clinical, pathological, and treatment-related factors influencing survival (15).

In this context, understanding the prognostic impact of variables such as LVI, PNI, pCR, neoadjuvant therapy response, surgical margin status, and postoperative CEA levels may improve patient counseling, follow-up planning, and adjuvant therapy decisions.

Furthermore, disease recurrence and mortality are not only influenced by tumor biology but also by treatment timing, emergency interventions, and patient-related factors such as BMI and age (16, 17). The increasing use of TNT in recent years adds further complexity to prognostication in rectal cancer (18).

Despite advances in treatment, real-world data from regional centers remain limited, particularly from Eastern Europe and the Middle East (19). There is a need for comprehensive survival and recurrence data from diverse populations to inform evidence-based practice.

This study was conducted in a high-volume tertiary hospital located in eastern Türkiye, aiming to analyze clinical, pathological, and treatment-related predictors of survival and recurrence in operated rectal cancer patients.

We evaluated the impact of demographic factors, neoadjuvant and adjuvant therapy, tumor characteristics, surgical outcomes, and biomarker levels on overall survival and disease-free survival.

Particular attention was given to the presence of LVI, PNI, response to neoadjuvant therapy, resection margin status, and follow-up metastasis.

We also analyzed the effect of TNT protocols and emergency surgery indications on long-term outcomes.

Survival curves were constructed using the Kaplan–Meier method, and independent predictors of mortality were identified using multivariate Cox regression.

By presenting a 10-year single-center experience, this study aims to contribute to the growing body of literature on rectal cancer outcomes in the context of evolving oncologic management.

To our knowledge, this is one of the most comprehensive analyses of rectal cancer surgery outcomes conducted in a regional center in Türkiye.

The results may have implications for patient selection, treatment planning, and postoperative follow-up strategies in similar settings.

## Materials and methods

### Study design and setting

This retrospective cohort study was conducted at the General Surgery Department of Van Training and Research Hospital, a tertiary care center located in eastern Türkiye. The study included patients who underwent curative-intent surgery for rectal cancer between January 2013 and December 2023. The institutional ethics committee approved the study protocol, and the study adhered to the principles of the Declaration of Helsinki.

### Patient selection

Patients diagnosed with rectal adenocarcinoma and who underwent elective or emergency surgical resection were included in the study. The inclusion criteria were:

- Histopathologically confirmed rectal adenocarcinoma
- Surgery performed with curative intent (LAR, APR, or segmental resection)
- Availability of complete clinical, pathological, and follow-up data
- Patients were excluded if they had:
- Synchronous or metachronous malignancies
- Palliative surgical interventions
- Incomplete follow-up or missing critical data

### Data collection

Data were obtained from hospital electronic records and pathology reports. Demographic variables (age, sex, height, weight, BMI), clinical presentation, tumor location, neoadjuvant and adjuvant therapies, radiologic and pathologic staging, operative details, and postoperative complications were recorded.

Histopathologic features including tumor differentiation, lymphovascular invasion (LVI), perineural invasion (PNI), pathologic T and N stage, number of retrieved and metastatic lymph nodes, resection margin status (R0/R1), and presence of complete pathological response were evaluated.

CEA levels were collected at the time of diagnosis, after neoadjuvant therapy, postoperatively, after adjuvant therapy, and at recurrence (if applicable). Tumor markers such as KRAS, NRAS, and BRAF mutations were also noted when available.

Pathologic response to neoadjuvant therapy was assessed according to the AJCC 8th edition tumor regression system. For the purposes of this analysis, pathologic complete response (pCR) was defined as the complete absence of viable tumor cells in both the primary rectal specimen and resected lymph nodes (ypT0N0). Because of the retrospective nature of the cohort and incomplete tumor regression grading in the early years, patients were categorized as having pCR or no pCR.

### Treatment modalities

Neoadjuvant treatments included long-course chemoradiotherapy (LCRT), short-course radiotherapy (SCRT), chemotherapy alone, and total neoadjuvant therapy (TNT). LCRT typically consisted of pelvic radiotherapy to a total dose of 45–50.4 Gy in 25–28 fractions, delivered with concurrent fluoropyrimidine-based chemotherapy (oral capecitabine 825 mg/m² twice daily on radiotherapy days or continuous-infusion 5-fluorouracil according to institutional protocols). SCRT consisted of 25 Gy in 5 fractions followed by surgery within the recommended interval.

Chemotherapy regimens included FOLFOX, XELOX, capecitabine, and 5-fluorouracil (5-FU). TNT was defined as the administration of systemic chemotherapy (most commonly FOLFOX or XELOX for X–Y cycles) in combination with LCRT or SCRT before surgery, with the intent to deliver all planned systemic therapy in the preoperative setting. Adjuvant chemotherapy and/or chemoradiotherapy was administered based on pathologic staging and multidisciplinary tumor board decisions.

### Follow-up and outcomes

Disease recurrence (local or distant) and mortality were recorded. Disease-free survival (DFS) was defined as the time from the date of curative-intent surgery to the first documented local or distant recurrence or death from any cause, whichever occurred first. Patients alive without evidence of recurrence at the last visit were censored at that date. Overall survival (OS) was defined as the time from the date of curative-intent surgery to death from any cause or last follow-up.

### Statistical analysis

Statistical analyses were performed using SPSS version 25.0 (IBM Corp., Armonk, NY, USA). Continuous variables were expressed as mean ± standard deviation, or median (minimum–maximum), and categorical variables as frequencies and percentages. Normality of distribution was assessed using the Kolmogorov–Smirnov test. Comparisons between groups were made using the Chi-square test for categorical variables, and the Mann–Whitney U test for non-parametric continuous variables.

Survival analysis was conducted using the Kaplan–Meier method and differences were evaluated with the log-rank test. Variables found to be significant in univariate analysis were included in a multivariate Cox regression model to identify independent predictors of mortality. A p-value <0.05 was considered statistically significant.

For multivariable analysis, variables that were statistically significant or showed a trend toward significance (p < 0.10) in univariate analyses and were considered clinically relevant (e.g. age, sex, LVI, PNI, emergency surgery, resection margin status, and development of metastasis during follow-up) were entered into a Cox proportional hazards model to identify independent predictors of mortality. During the study period, a total of 22 mortality events were observed. In the final multivariable Cox proportional hazards model, two covariates were included, resulting in an event-per-variable (EPV) ratio of 11. This EPV exceeds the commonly recommended minimum threshold of approximately 10, thereby reducing but not completely eliminating the risk of model overfitting. Accordingly, the results of the multivariable analysis should be interpreted as exploratory and hypothesis-generating rather than definitive. The proportional hazards assumption was assessed using log–log survival plots and Schoenfeld residuals and was not violated. The ratio of events to covariates was checked to reduce the risk of model overfitting.

Variables with p < 0.10 in univariate analysis and those considered clinically relevant (age, sex, LVI, PNI, emergency surgery, resection margin status, CEA levels, and metastasis during follow-up) were included in the multivariate Cox regression model.

Collinearity was assessed using variance inflation factors (VIFs) and pairwise correlation matrices; variables showing collinearity were evaluated and the most clinically meaningful variable retained.

The proportional hazards assumption was tested with Schoenfeld residuals and log–log plots, showing no violations.

Because survival data were censored, survival outcomes were summarized using median survival times with 95% confidence intervals, and mean survival values were not reported.

Given the limited number of mortality events observed in the cohort (n = 22), particular attention was paid to the event-per-variable (EPV) ratio in multivariable modeling. To minimize the risk of model overfitting, the number of covariates included in the Cox proportional hazards regression was restricted, and only variables that were both clinically relevant and statistically significant in univariate analyses were entered into the multivariable model. The EPV ratio was carefully considered during model construction, in line with commonly accepted biostatistical recommendations suggesting a minimum EPV of approximately 10 for reliable estimates.

Because of the retrospective design and the fixed sample size of the study, no formal *a priori* sample size or power calculation was performed. The study population represents all consecutive eligible patients treated during the study period. Therefore, the analyses should be interpreted as exploratory, and the results, particularly from multivariable models, should be considered hypothesis-generating rather than definitive.

## Results

### Patient demographics and tumor characteristics

A total of 122 patients were included in the study. The mean age was 60.7 ± 11.8 years (median, 61.4; range, 30.6–84.5). Of the cohort, 55 patients (45.1%) were female and 67 (54.9%) were male. The most common presenting symptoms were rectal bleeding (59.8%) and changes in bowel habits (48.4%), whereas pain (4.9%) and weight loss (0.8%) were less frequent. Tumors were located in the distal rectum in 36.1% of patients, the middle rectum in 45.9%, and the proximal rectum in 18.0% (**Table 1**).

**Table 1:** Patient demographic and clinical characteristics, and tumor localization.

Variable	Count (n)	Percentage (%)
Sex		
Female	55	45.1
Male	67	54.9
Complaint		
Rectal bleeding	73	59.8
Defecation difference	59	48.4
Pain	6	4.9
Lose weight	1	0.8
Tumor localization		
Distal rectum	44	36.1
Middle rectum	56	45.9
Proximal rectum	22	18.0

Lymphovascular invasion (LVI) was identified in 27.0% of patients and perineural invasion (PNI) in 35.2%. Microsatellite instability (MSI) positivity was detected in 25.4% of cases. Histopathologic grading revealed well-differentiated tumors in 29.5%, moderately differentiated tumors in 56.6%, and poorly differentiated tumors in 13.9% of patients. Clinically, most tumors were staged as T3 (55.7%) or T4 (38.5%), and lymph node positivity was present in 77% of patients. According to clinical TNM classification, stage III disease predominated (79.5%). Neoadjuvant therapy was administered to 77% of the cohort (**Table 2**).

**Table 2:** Tumor characteristics and clinical staging.

Variable	Count (n)	Percentage (%)
LVI	33	27.0
PNI	43	35.2
MSI	31	25.4
Differentiation		
Good (gr 1)	36	29.5
Mid (gr 2)	69	56.6
Poor (gr 3)	17	13.9
cT stage		
T2	7	5.7
T3	68	55.7
T4	47	38.5
cN stage		
Negative	28	23
Positive	94	77
Clinic TNM at diagnosis		
TNM 1	1	0.8
TNM 2	24	19.7
TNM 3	97	79.5

Among patients receiving neoadjuvant treatment, long-course radiotherapy was the most common modality (51.1%), followed by short-course radiotherapy (3.2%) and chemotherapy alone (1.1%). Total neoadjuvant therapy (TNT) was applied in 44.7% of patients, and among these, 73.8% received radiotherapy following chemotherapy. The most frequently used chemotherapy regimens were capecitabine (38.3%), XELOX (36.2%), FOLFOX (20.2%), and 5-fluorouracil (5.3%).

### Surgical and pathological outcomes

Elective surgery was performed in 89.3% of patients, whereas 10.7% underwent emergency surgery, most commonly due to ileus (76.9%), followed by perforation (15.4%) and bleeding (7.7%). Surgical procedures included low anterior resection in 77.0% of cases, abdominoperineal resection in 22.1%, and segmental resection in 0.8%.

Pathologic evaluation demonstrated T3 tumors in 45.1% of patients, T2 tumors in 25.4%, T4 tumors in 10.7%, and T1 tumors in 2.5%, while a complete pathologic response (T0) was achieved in 16.4%. Pathologic nodal staging revealed N0 disease in 71.3% of patients, N1 in 23.0%, N2 in 4.9%, and N3 in 0.8%, with metastatic lymph nodes identified in 27% of cases. R0 resection was achieved in 95.9% of patients, whereas 4.1% had microscopic residual disease (R1). A pathologic complete response (ypT0N0) was observed in 18 patients (14.8%).

### Adjuvant treatment and genetic markers

Adjuvant chemotherapy was administered to 87.7% of patients, predominantly using CAPOX (55.1%) and FOLFOX (39.3%) regimens. Adjuvant chemoradiotherapy was given to 20.5% of patients, most commonly with capecitabine-based protocols. Molecular analysis was available in a limited subgroup of 10 patients, among whom KRAS, NRAS, and BRAF mutations were each detected in 40%.

### Mortality analysis

**Figure 1:**
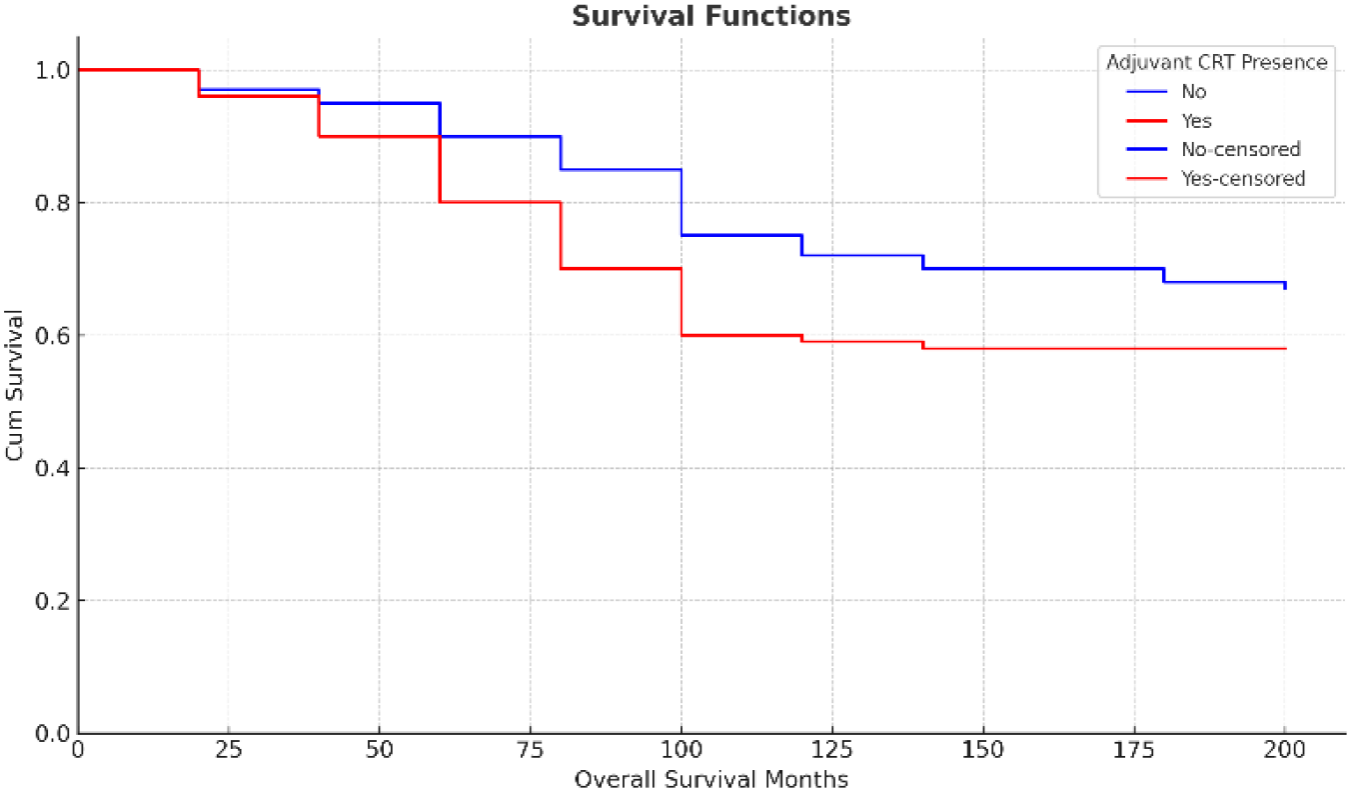
Kaplan-Meier survival curves according to the presence of adjuvant chemoradiotherapy (CRT).

During the follow-up period, 22 patients (18%) died. Mortality was significantly associated with adverse clinicopathologic features, including pain at presentation, presence of LVI and PNI, poor tumor differentiation, clinical T4 stage, emergency surgery, R1 resection, and the development of metastasis during follow-up. Treatment-related factors associated with mortality included lower rates of neoadjuvant therapy and higher use of adjuvant chemoradiotherapy (**Figure 1**). In addition, higher body weight, increased body mass index, larger tumor size, a greater number of metastatic lymph nodes, and elevated CEA levels at multiple time points were significantly associated with mortality. Receiver operating characteristic (ROC) analysis demonstrated that preoperative carcinoembryonic antigen (CEA) levels had a fair discriminative ability for predicting mortality (AUC = 0.673) (**Figure 3**).

**Figure 2:**
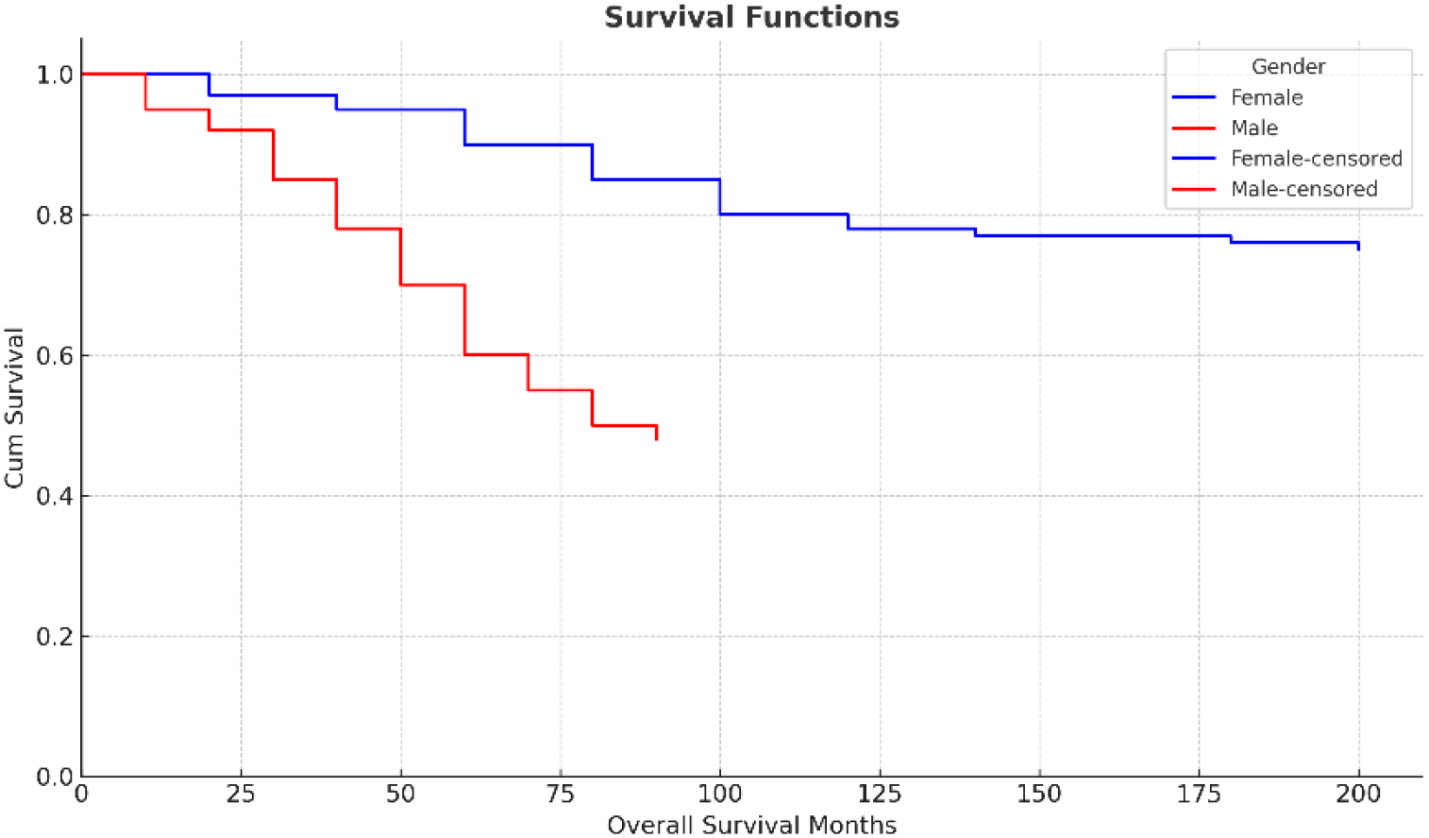
Kaplan-Meier survival curves based on gender.

**Figure 3:**
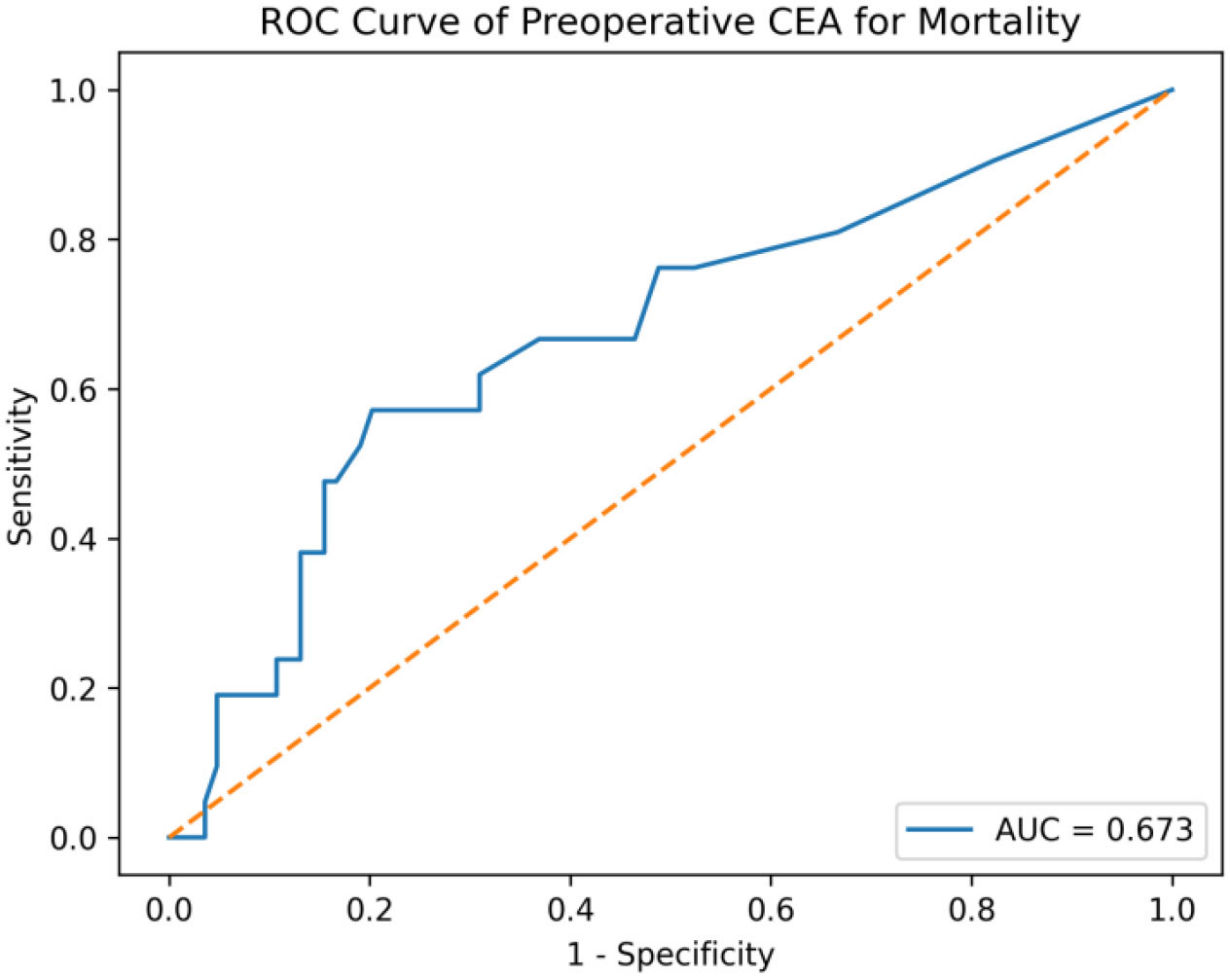
Receiver operating characteristic (ROC) curve of preoperative carcinoembryonic antigen (CEA) for prediction of mortality.

### Recurrence analysis

**Figure 4:**
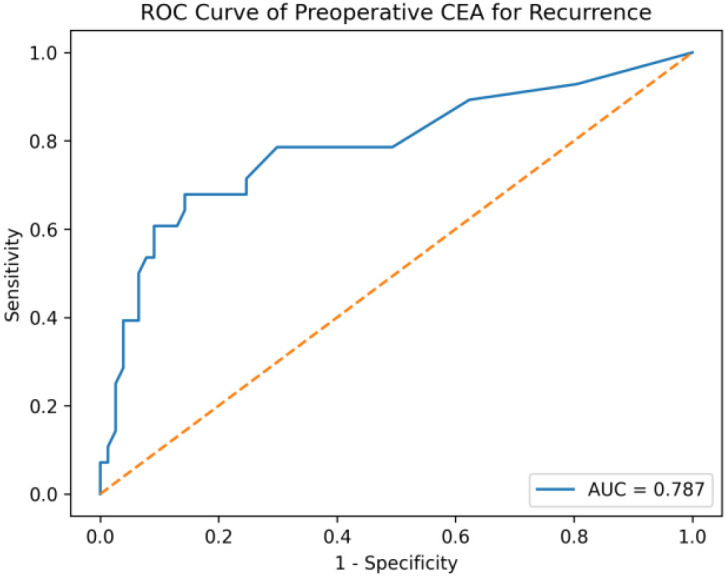
Receiver operating characteristic (ROC) curve of preoperative carcinoembryonic antigen (CEA) for prediction of disease recurrence.

Tumor recurrence occurred in 27 patients (22.1%). Recurrence was significantly more frequent in patients with middle rectal tumors, LVI, PNI, poor tumor differentiation, advanced clinical and pathological T stage, nodal involvement, stage III disease, R1 resection, and the presence of metastatic lymph nodes. Quantitative factors associated with recurrence included higher body weight, increased BMI, greater visceral adiposity area, larger tumor size, a higher number of metastatic lymph nodes, and elevated CEA levels at various follow-up points. These findings indicate that both aggressive tumor biology and patient-related metabolic factors contribute to recurrence risk. Receiver operating characteristic (ROC) analysis demonstrated that preoperative carcinoembryonic antigen (CEA) levels had a fair discriminative ability for predicting mortality (AUC = 0.673). ROC analysis showed that preoperative CEA had good predictive performance for disease recurrence, with an area under the curve (AUC) of 0.787(**Figure 4**).

### Overall survival and disease-free survival

**Figure 5:**
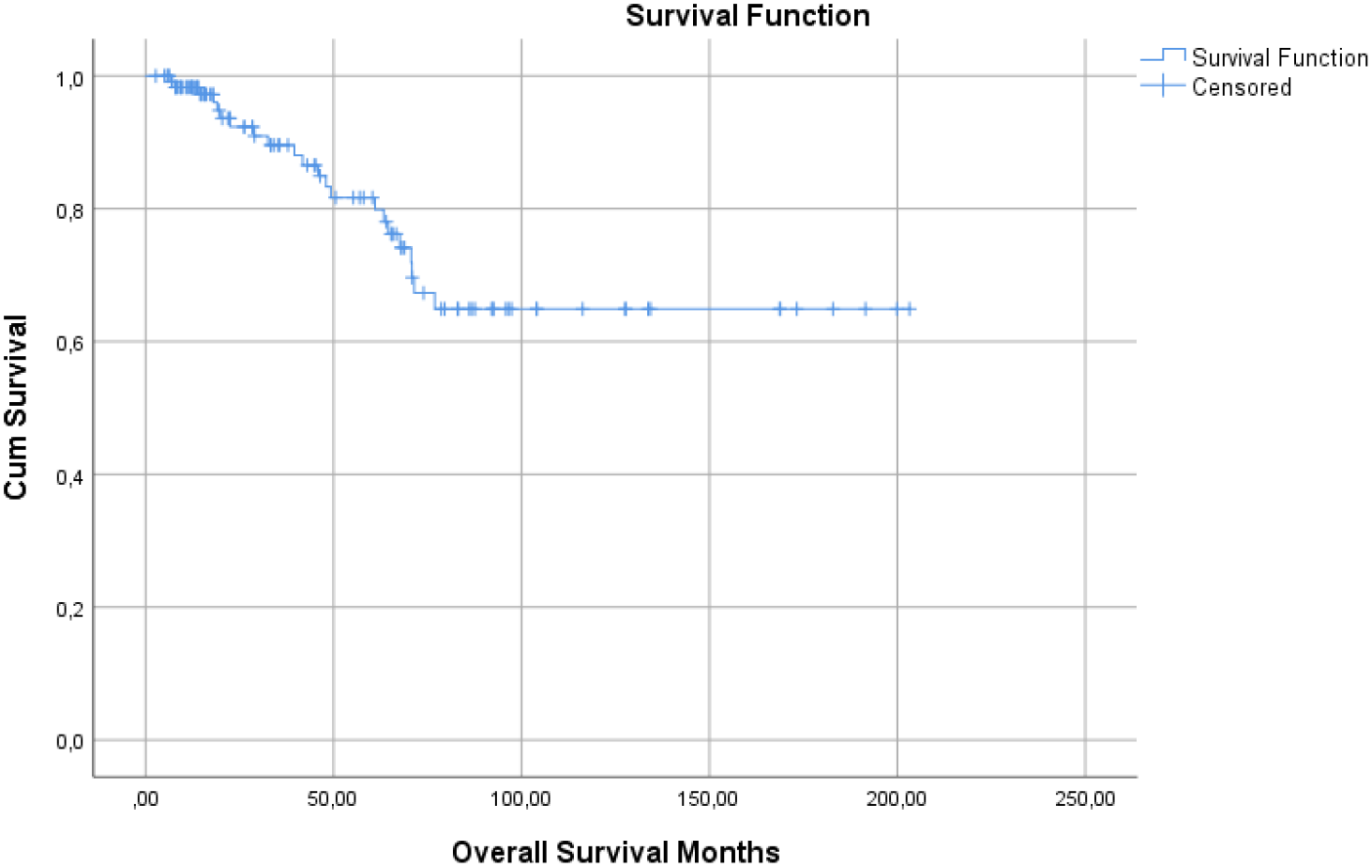
Overall survival analyse.

The median postoperative overall survival was 156.0 months (95% CI, 132.4–179.6), with an estimated 5-year overall survival rate of 78.4% (**Figure 5**). Overall survival was significantly shorter in patients who developed metastasis during follow-up, as well as in those with LVI and PNI. In multivariate Cox regression analysis, follow-up metastasis (OR = 8.753, p < 0.001) and LVI (OR = 3.889, p = 0.016) emerged as independent predictors of mortality (**Table 3**).

**Table 3:** Multivariate Cox regression analysis of factors affecting mortality.

Variable	p	Hazard Ratio	%95 CI	
			Min	Max
Gender (Male vs Female)	0,194	1,947	0,712	5,327
Metastasis during follow-up (Yes vs No)	<0,001**	8,753	3,087	24,818
LVI (Present vs Absent)	0,016*	3,889	1,289	11,735
PNI (Present vs Absent)	0,179	,430	0,125	1,472

*p<0,05, **p<0,01.

Bold values indicate statistical significance (p < 0.05; p < 0.01).

The median disease-free survival was 28.5 months (95% CI, 22.0–36.5), with an estimated 5-year disease-free survival rate of 63.4%. Disease-free survival was significantly shorter in patients with N2/3 nodal stage and in those with LVI. Differences in disease-free survival across clinicopathologic subgroups were evaluated using Kaplan–Meier analysis and log-rank tests.

## Discussion

In this retrospective cohort study conducted at a tertiary center in eastern Türkiye, we evaluated factors influencing survival and recurrence in patients who underwent curative surgery for rectal cancer. Our findings highlight the prognostic impact of lymphovascular invasion (LVI), perineural invasion (PNI), tumor differentiation, margin status, and recurrence during follow-up.

The overall survival rate and disease-free survival durations observed in our cohort are consistent with previously reported results from high-volume centers, despite geographic and demographic differences (20).

We observed significantly lower survival in male patients compared to females, in line with prior research showing gender-based survival disparities in colorectal cancer (21) (**Figure 2**). Several biological and hormonal factors have been suggested to contribute to this difference, although the exact mechanisms remain unclear.

LVI emerged as one of the strongest predictors of both recurrence and mortality. Patients with LVI had significantly reduced overall and disease-free survival, even after adjusting for other variables in multivariate analysis. This finding aligns with multiple prior studies reporting LVI as a critical histopathologic marker of aggressive disease (22, 23).

Similarly, PNI was significantly associated with poor survival and high recurrence rates. PNI represents tumor spread along nerve sheaths, a pathway facilitating local invasion and distant metastasis (24). Its presence often indicates a higher risk of systemic disease, supporting its inclusion in routine pathology reports.

The association between elevated BMI/visceral adiposity and recurrence may reflect the biological interplay between metabolic dysregulation and tumor progression. Adipose-tissue–derived cytokines, insulin resistance, and chronic inflammation may promote tumor invasion and metastatic potential. These mechanisms may partly explain why patients with higher metabolic burden demonstrated worse DFS in our cohort (21).

Poor tumor differentiation was another independent risk factor for worse outcomes. Poorly differentiated tumors were associated with higher rates of both recurrence and mortality, corroborating established evidence linking histologic grade to aggressive biological behavior (25).

Our study also identified resection margin status as a key determinant of prognosis. Patients with R1 resection (microscopically positive margins) had significantly higher recurrence and mortality rates compared to those with R0 resections. This emphasizes the importance of achieving clear margins during rectal cancer surgery (26).

The development of metastasis during follow-up was the most powerful predictor of mortality in our cohort. Patients who developed distant metastases had an 8.75-fold increased risk of death, underscoring the need for vigilant surveillance and timely systemic therapy in high-risk patients.

The number of metastatic lymph nodes and advanced pathologic N stage (N2/3) were also significantly associated with worse outcomes. This is in accordance with the TNM staging system, where nodal burden is a principal prognostic factor (11).

Preoperative and postoperative CEA levels were significantly higher in patients who experienced recurrence or died, supporting the role of CEA as a dynamic biomarker for prognosis and monitoring (13). In particular, persistently elevated postoperative or post-adjuvant CEA levels should prompt closer surveillance.

Notably, patients who underwent emergency surgery had significantly higher mortality. Emergency indications such as obstruction and perforation are known to adversely affect oncologic outcomes due to inflammation, delayed diagnosis, and less optimal preoperative preparation (16). While emergency presentation may reflect biologically aggressive disease, poor outcomes in this subgroup also likely relate to systemic deterioration, lack of preoperative staging, absence of neoadjuvant therapy, and increased perioperative complication risk. Stratified interpretation is therefore required to distinguish between disease-driven and care-delivery–related factors.

Neoadjuvant therapy, including total neoadjuvant therapy (TNT), has been associated with improved local control and survival in recent randomized trials (27, 28). In our retrospective series, patients receiving neoadjuvant treatment tended to have better outcomes, but the study was not specifically powered to demonstrate a statistically significant survival advantage of TNT over conventional chemoradiotherapy. Therefore, our conclusions focus primarily on the prognostic impact of clinicopathologic variables such as LVI, PNI, margin status, and nodal involvement.

Pathologic complete response (pCR) was observed in 14.8% of our patients, a rate comparable to previous series using TNT protocols (29). Although pCR was not independently associated with survival in our study, prior studies have shown that patients with pCR have excellent long-term outcomes (30).

Our study also confirmed that larger tumor size and higher BMI were associated with poor outcomes. Obesity may contribute to tumor progression through inflammatory, hormonal, and immune-modulating mechanisms, although its role in rectal cancer prognosis remains debated (31).

Middle rectum tumors were more commonly associated with recurrence in our cohort. While tumor location within the rectum has been studied as a prognostic variable, results across studies have been heterogeneous (32).

The use of adjuvant chemoradiotherapy was more common in patients with poor prognostic features. Although this may reflect selection bias, it also supports the notion that aggressive treatment is often required for high-risk patients.

Despite advancements in multimodal therapy, a significant proportion of patients continue to experience recurrence. This suggests the need for more refined risk stratification tools, potentially incorporating molecular and genetic profiling.

KRAS, NRAS, and BRAF mutations were not widely available in our series but remain promising biomarkers for prognosis and treatment response, especially in the metastatic setting (33).

Our findings are broadly consistent with national colorectal cancer data reported from other tertiary centers in Türkiye, particularly regarding recurrence rates and the prognostic impact of LVI and nodal status. This alignment suggests that despite regional resource constraints, our cohort reflects the general disease pattern observed nationwide (19).

Our study’s strengths include a long follow-up period, real-world population, and comprehensive evaluation of multiple prognostic factors. Importantly, the study was conducted in a regional tertiary hospital, reflecting outcomes in non-metropolitan settings.

However, the study is not without limitations. Its retrospective design introduces potential bias and confounding. Moreover, the sample size, although sufficient for several comparisons, may limit the power for subgroup analyses. In addition, treatment patterns in our center, including a relatively high use of adjuvant chemotherapy and lower use of standardized neoadjuvant protocols in earlier years, reflect institutional practice and referral patterns and may limit the generalizability of our results to other settings. A further limitation is the low availability of molecular profiling (KRAS, NRAS, BRAF), particularly in the earlier years of the cohort. As molecular markers increasingly influence prognosis and treatment decisions, future studies with comprehensive genomic data are warranted.

Some variables, such as MSI status and molecular markers, were not available for all patients, limiting the ability to assess their prognostic role. Additionally, treatment decisions were made over a decade, during which protocols evolved, potentially influencing results.

Nevertheless, our findings highlight several modifiable and non-modifiable predictors of recurrence and mortality after rectal cancer surgery. Achieving negative margins, delivering effective neoadjuvant treatment, and monitoring CEA trends may improve outcomes.

Future research should focus on integrating clinical, pathological, and molecular data to create personalized risk models. Prospective studies from similar tertiary centers in developing regions are also needed to validate and extend our findings.

In conclusion, our study underscores the prognostic value of LVI, PNI, tumor grade, nodal status, margin status, and follow-up metastasis in rectal cancer patients undergoing curative surgery. Regular CEA monitoring and appropriate use of neoadjuvant therapy may improve outcomes in high-risk groups.

By identifying key risk factors, this study may help clinicians tailor follow-up and adjuvant therapy strategies to individual patients. These insights are particularly relevant for surgeons and oncologists working in resource-limited settings.

## Data Availability

The raw data supporting the conclusions of this article will be made available by the authors, without undue reservation.
